# Prognostic importance of PTEN, EGFR, HER-2, and IGF-1R in gastric cancer patients treated with postoperative chemoradiation

**DOI:** 10.3906/sag-1802-34

**Published:** 2019-08-08

**Authors:** Berrin BENLİ YAVUZ, Mehmet KOÇ, Sümeyye KOZACIOĞLU, Gül KANYILMAZ, Meryem AKTAN

**Affiliations:** 1 Department of Radiation Oncology, Faculty of Medicine, Necmettin Erbakan University, Konya Turkey; 2 Department of Pathology, Faculty of Medicine, Necmettin Erbakan University, Konya Turkey

**Keywords:** Gastric cancer, PTEN, EGFR, HER-2, IGF-1R, radiotherapy

## Abstract

**Background/aim:**

This study aimed to describe the prognostic importance of epidermal growth factor (EGFR), phosphatase and tensin homolog (PTEN), human EGF receptor-2 (HER-2), and insulin-like growth factor 1 receptor (IGF-1R) in gastric cancer patients treated with postoperative chemoradiation therapy.

**Materials and methods:**

Sixty-nine patients treated with adjuvant chemoradiation therapy were retrospectively evaluated. Tumor samples were stained immunohistochemically.

**Results:**

All patients were treated with 3D conformal radiation therapy with concomitant and adjuvant chemotherapy. Perineural invasion (PNI) (P = 0.042), prechemoradiation therapy albumin levels below 3.5 mg/dL (P = 0.011), and EGFR positivity (P = 0.008) had negative effects on overall survival (OS). The median OS was 26 months for patients with PNI (+), 34.9 months for those with PNI (–), 19.5 months for those with albumin levels below 3.5 mg/dL, and 33.2 months for those with albumin levels above 3.5 mg/dL. IGF-1R (+) (P = 0.035) and history of cigarette smoking (P = 0.033) were observed to have a statistically significantly negative effect on disease-free survival (DFS). The median DFS was 29.2 months for IGF-1R (+) patients, 37.9 months for those with IGF-1R (-), and 26.3 and 40.59 months for smokers and nonsmokers, respectively.

**Conclusion:**

IGF-1R and EGFR may be used for patient selection in future prospective studies that evaluate the prognostic importance of these receptors.

## 1. Introduction

Gastric cancer is the second leading cause of cancer-related mortality worldwide with the highest mortality rates being reported in East Asia [1]. As it is diagnosed at an advanced stage of the disease, the 5-year survival rate of patients with gastric cancer is lower than 20% [2]. The main treatment for gastric cancer is surgery [1]. Although surgery is the only possible curative treatment, the distant and regional recurrence rates of postresection curative treatment are still high. Consequently, adjuvant therapies such as radiation therapy (RT), chemotherapy (CT), or chemoradiation therapy (CRT) have been studied in patients with gastric cancer after surgical resection [3]. Neoadjuvant or adjuvant therapy is recommended in patients at stage 2 and those at advanced stages or in node-positive patients, which might improve the treatment outcomes. The Intergroup 0116 study, a randomized phase 3 trial, compared observation versus adjuvant chemoradiation therapy. This study showed the advantages of combined adjuvant therapy on overall survival (OS) [4]. Prognosis for patients with locally advanced stage disease is poor despite multimodal therapy. Consequently, new strategies must be developed to improve the outcomes. 

The most significant prognostic indicator for gastric cancer is tumor stage. Lymph node involvement and the number and localization of lymph nodes are also important risk factors [5]. Particular attention should be given to the biomarkers that might assist in defining subgroups of the individual therapies [6]. These novel prognostic factors include phosphatase and tensin homolog (PTEN), epidermal growth factor (EGFR), human EGF receptor-2 (HER-2), and insulin-like growth factor 1 receptor (IGF-1R). EGFR is localized on the external cell surface and is a member of the tyrosine kinase (TK) receptor family. EGFR overexpression is observed in various human malignancies including lung, breast, colon, and gastric cancers [7]. EGFR has been shown to be a dependent prognostic factor in numerous types of cancers. The clinical role of EGFR in gastric cancer is not clear. HER-2 is a transmembrane TK receptor [8,9]. Intestinal-type and well-differentiated gastric cancers have a higher rate of HER-2 positivity than poorly differentiated and diffused-type gastric cancers [7]. The overexpression of HER-2 and EGFR is associated with poor prognosis and has been reported in approximately 30%–50% of gastric cancers [10,11]. *PTEN* is a tumor-suppressor gene localized on chromosome 10 [12]. Studies have reported that inactivation of the *PTEN* gene is closely associated with the progression and incidence of gastric cancer [13]. The PTEN protein affects apoptosis, migration, metabolism, transcription, and translation and also inhabits cellular invasion and metastasis [14]. Due to genetic modification, *PTEN* gene inactivation might be observed in endometrial carcinoma, glioblastoma multiforme, prostate, skin, and breast cancers [3]. IGF-1R is a membrane cell receptor that is frequently overexpressed in human cancers [11]. IGF-1R has a role in cellular proliferation, malignant transformation, differentiation, and prevention of apoptosis [15]. There is a relationship between IGF-1R and survival in breast cancer and solid tumors, but IGF-1R expression in gastric cancer is poorly understood. IGF-1R expression is a predictor for poor results [11]. The overexpression of IGF-1 was distinct in large tumors with high mitotic activity, and it was correlated with high risk of malignancy. The overexpression of IGF-1R was correlated with the overexpression of IGF, high mitotic activity, and high risk of malignancy [16]. 

The present study was designed to define the prognostic importance of HER-2, IGF-1R, PTEN, and EGFR in gastric cancer patients treated with postoperative chemoradiation therapy. 

## 2. Materials and methods

The hospital records of 69 patients with nonmetastatic gastric cancer were retrospectively evaluated between January 2010 and October 2013. The study was approved by the Ethics Committee of Necmettin Erbakan University. Paraffin-embedded tumor samples were immunohistochemically stained for PTEN, EGFR, HER-2, and IGF-1R. All patients were treated with 3D conformal radiation therapy with concomitant and adjuvant chemotherapy. Formalin-fixed paraffin-embedded (FFPE) tumor samples were immunohistochemically stained for PTEN, EGFR, HER-2, and IGF-1R. Sections of tumor blocks of 4 µm were stained with hematoxylin and eosin, and the samples were evaluated by a pathologist. All reagents and equipment for immunohistochemistry (IHC) were manufactured by Ventana Medical Systems (Tucson, AZ, USA). FFPE sections were stained automatically with Ventana Benchmark XD by using primary antibodies directed against EGFR, PTEN, HER-2, and IGF-1R. 

A scoring system based on the variability of staining intensity was used. Brown membranous staining was considered positive for HER-2. The results were evaluated as follows: 0, no staining or membranous reactivity in <10% of cells; (+), faintly to barely perceptible membranous reactivity in >10% of cells; (++), basolateral membranous staining of weak to moderate intensity in >10% of cells; and (+++), basolateral membranous staining of strong intensity in >10% of cells [9]. EGFR and IGF-1R tumor cell membrane immunostaining was scored as 0, 1+ , 2+, or 3+. The results were evaluated as follows: 0, no membranous reactivity within the tumor; 1+, 2+, or 3+, depending on the intensity above the background level [1,15]. For EGFR, IGF-1R, and HER-2, an IHC score of 3+ was defined as positive. The intensity of staining for PTEN was scored using a 0 to 3 grading system: 0, negative; 1, weak; 2, moderate; and 3, strong. The level of PTEN staining was evaluated by calculating the immunoreactive score (IRS) from the staining intensity and the percentage of PTEN-positive cells [17]. IRS = 0 and IRS = 2–3 were considered as negative and positive, respectively. The total PTEN expression was calculated as cytoplasmic PTEN expression + nuclear PTEN expression. 

### 2.1. Statistical analysis 

Statistical analysis was performed using SPSS 16.0 (SPSS Inc., Chicago, IL, USA). Primary and secondary outcomes were OS and disease-free survival (DFS). The time from diagnosis to the date of the patient’s death or last follow-up was defined as OS. The time from diagnosis to the date of the documented progression, distant metastases, or recurrence was defined as DFS. Survival was calculated with the Kaplan–Meier method and a log-rank test. Fisher’s exact test was used to analyze HER-2, IGF-1R, PTEN, and EGFR (positive or negative) and univariate correlations between clinicopathological variations. Multivariate analysis of the data was performed using the Cox proportional hazards model. P < 0.05 was accepted as statistically significant. 

## 3. Results

### 3.1. Patient and tumor characteristics

**Figure 1:**
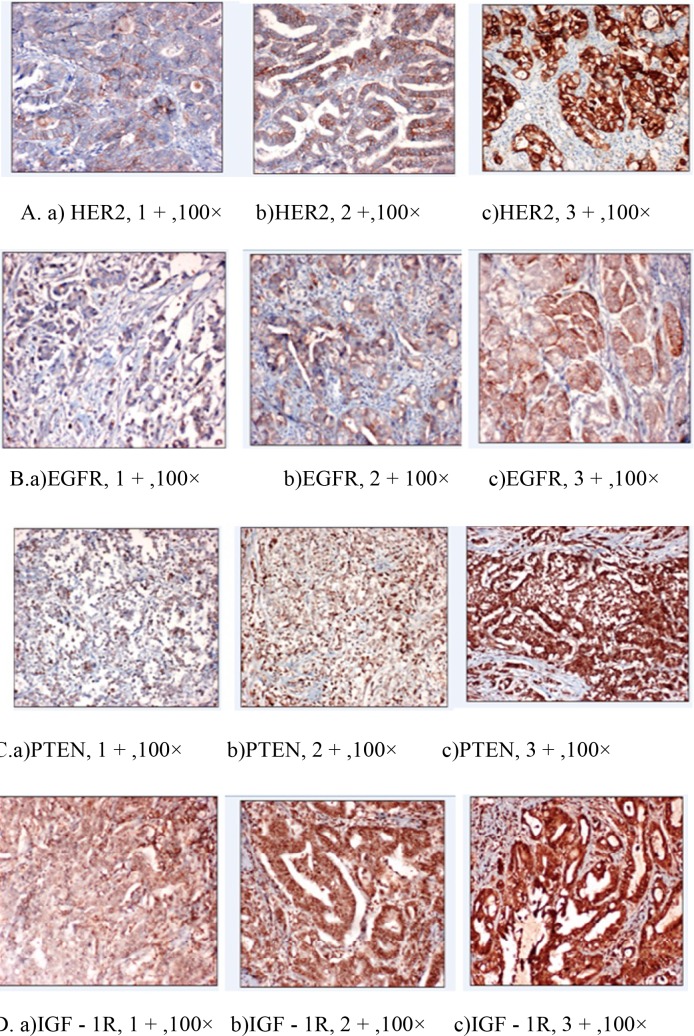
Typical examples of positive immunohistochemical staining for HER-2 (A), EGFR (B), PTEN (C), and IGF-1R (D).

Of the patients, 72.5% were male and 27.5% were female. The median age was 59 years (range: 34–80 years), and 65.2% were smokers. Karnofsky performance scores of all patients were above 70. The median follow-up period was 15.9 months (range: 3.2–44.68 months). Tumors were localized in the cardia, antrum, corpus, and fundus of 27.5%, 31.9%, 39.1%, and 1.4% of the patients, respectively. The median period between surgery and RT was 68 days (range: 34–284 days). D2 dissection was implemented in 82% of patients. All patients were treated with 3D conformal radiation therapy with concomitant and adjuvant chemotherapy. In 31.9% (n = 22) of the patients, inadequate lymph node dissection was performed, and 7 of these patients were stage 1. The radiation dose for postoperative adjuvant therapy was 45–50.4 Gy. Adjuvant chemotherapy was administered for 5.8% (n = 4) of patients with TCF, 63.7% (n = 44) with FUFA, and 30.4% (n = 21) with CF. The median serum albumin level was 3.8 mg/dL prior to treatment (range: 2.4–3.8 mg/dL). Histopathological examination revealed adenocarcinoma in 75.7% (n = 53) of patients and signet-ring cell adenocarcinoma in 24.3% (n = 16) of patients (Table 1). There was no statistically significant difference in EGFR and PNI in stage 1, 2, and 3 (Table 2). Distant metastasis was observed in 12 patients and local recurrence was not defined. HER-2, EGFR, IGF-1R, and PTEN were positive in 9 (13%), 10 (14.5%), 24 (34.8%), and 45 (65.2%) patients, respectively (Figure 1).

**Table 1:** Patient and tumor characteristics.

	n (%)
SexMaleFemale	50 (72.5%)19 (27.5%)
Cigarette smoking	45 (65.2%)
Primary location of tumorCardiaAntrumCorpusFundus	19 (27.5%)22 (31.9%)27 (39.1%)1 (1.4%)
Type of gastrectomyTotal Subtotal	30 (43.5%)39 (56.5%)
Histopathological typeAdenocarcinomaSignet ring cell adenocarcinoma	53 (75.7%)16 (24.3%)
Lymphovascular invasion	32 (46.4%)
Perineural invasion	43 (62.3%)
StageStage 1Stage 2Stage 3	7 (10.1%)12 (17.4%)50 (72.5%)
	Median (range)
Age, years	59 (34–80)
Follow-up period, months	15.9 (3.2–44.68)
Period between surgery and RT, days	68 (34–284)
Pretreatment albumin, g/dL	3.8 (2.4–3.8)
Tumor size, cm	1–16
Number of resected lymph nodes	20 (4–52)
Number of involved lymph nodes	2 (0–34)

**Table 2:** EGFR and PNI distribution according to stages.

	Stage 1	Stage 2	Stage 3	P-value
PNI				
Negative	3 (11.5%)	3 (11.5%)	20 (76.9%)	0.602
Positive	4 (9.3%)	9 (20.9%)	30 (69.8%)	
EGFR				
Negative	6 (10.2%)	11 (18.6%)	42 (71.2%)	0.795
Positive	1 (10%)	1 (10%)	8 (80%)	

### 3.2. Survival analysis

**Figure 2:**
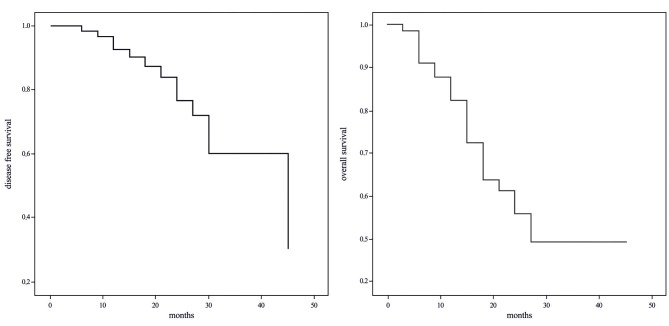
Kaplan-Meier curves for overall survival and disease-free survival.

The median OS was 41 months. The overall survival rates at 1, 2, and 3 years were 88%, 56%, and 49%, respectively. The 1- , 2-, and 3-year DFS rates were 94%, 78%, and 61%, respectively (Figure 2). 

**Figure 3:**
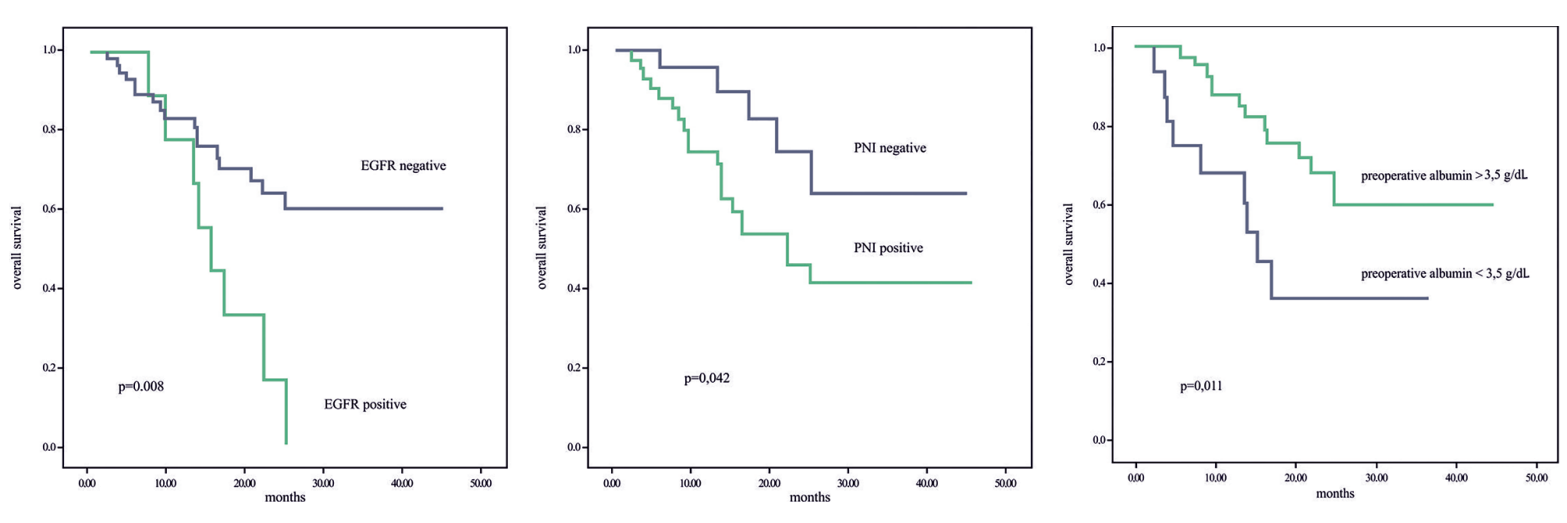
Relationships between overall survival and PNI, EGFR expression, and preoperative albumin.

Perineural invasion (PNI) (log-rank; P = 0.042), prechemoradiation therapy albumin level below 3.5 mg/dL, and EGFR positivity were found to have negative effects on OS. The median OS was 26 months for patients with PNI (+), 34.9 months for those with PNI (–), 19.5 months for those with albumin levels of <3.5 mg/dL, and 33.2 months for those with albumin levels of >3.5 mg/dL. OS was 16.2 and 32.1 months in EGFR (+) and EGFR (-) patients, respectively (P = 0.008) (Figure 3).

**Table 3:** Cox regression multivariate analysis of prognostic factors for OS (a) and DFS (b) in all patients.

	P-value	95% CI
HER-2	0.238	0.313–1.651
IGF-1R	0.033	1.095–8.355
EGFR	0.477	0.335–10.35
PTEN	0.943	0.451–2.355
Lymph node	0.038	0.576–2.902
Smoking	0.282	0.314–1.401

	P -value	95% Cl
HER-2	0.959	0.000–2.448
IGF-1R	0.039	0.001–0.909
EGFR	0.038	0.006–2.944
PTEN	0.789	0.110–9.512
Lymph node	0.769	0.056–49.05
Smoking	0.694	0.000–5.688

**Figure 4:**
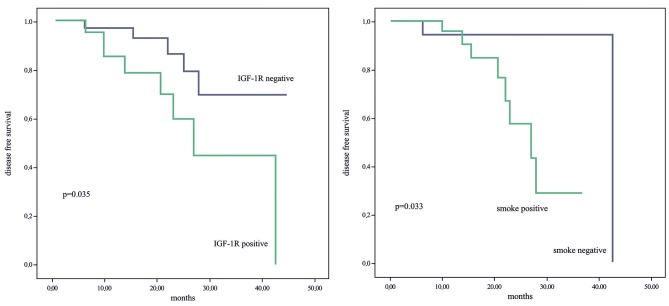
Relationships between disease-free survival and IGF-1R (a) and smoking (b).

In univariate analyses, PNI (+) (P = 0.038), albumin level <3.5 mg/dL (P = 0.035), existence of lymph node involvement (P = 0.035), and EGFR (+) (P = 0.003) had negative effects on prognosis. However, in multivariate analyses, only IGF-1R (+) (P = 0.033) and lymph node involvement (P = 0.038) had poor prognostic effects on OS (Table 3a). IGF-1R (+) (log-rank; P = 0.035) and history of smoking (log-rank; P = 0.033) were observed to have a statistically significantly negative effect on DFS. The median DFS was 29.2 months for IGF-1R (+) patients, 37.9 months for those with IGF-1R (-), and 26.3 and 40.59 months for smokers and nonsmokers, respectively. In univariate analyses, IGF-1R positivity was found to be a poor prognostic factor for DFS (P = 0.024). In multivariate analyses, IGF-1R positivity (P = 0.039) and EGFR (+) (P = 0.038) had positive prognostic effects on DFS (Figure 4) (Table 3b).

## 4. Discussion

In our study, 24 of 69 cases (34.8%) of gastric cancer were positive for IGF-1R expression. We demonstrated that IGF-1R expression was correlated with prognosis and OS in gastric cancer. IGF-1R positivity had a prognostic effect on DFS in both univariate and multivariate analysis. The positive EGFR expression rate was 14.5% in gastric cancer tissue. EGFR positivity had a negative impact on OS. The median OS rates were 16.2 and 32.1 months in EGFR (+) and EGFR (-) patients, respectively (P = 0.008). In multivariate analysis, EGFR positivity had a negative effect on DFS (P = 0.038). In our study, HER-2 and PTEN were not prognostic factors in gastric cancer patients. 

The correlation between IGF-1R expression and outcome was shown for breast and other solid tumors. However, the role of IGF-1R expression is not clearly defined in gastric cancers. Matsubara et al. showed that IGF-1R expression was a significant independent predictor of poor outcomes. However, EGFR and HER-2 were not related to OS [11,18].

EGFR is the most important member of the growth factor receptor family. EGFR expression was found to be related to survival in gastric cancer patients [6,11,18,19]. Atmaca et al. reported similar median OS in patients with positive EGFR compared to those with negative tumors [6]. Zhang et al. detected a positive correlation between high EGFR expression and cetuximab response in gastric cancer [20]. Cetuximab, lapatinib, and panitumumab are anti-EGFR inhibitors. The EXPAND study, a randomized phase 3 trial, compared capecitabine plus cisplatin alone versus cisplatin + capecitabine + cetuximab therapy. The median progression-free survival (PFS) and OS were 5.6 and 10.7 months, respectively, in the capecitabine plus cisplatin group compared to 4.4 and 9.4 months in patients receiving capecitabine plus cisplatin and cetuximab (P = 0.315 and P = 0.954). The addition of cetuximab to capecitabine + cisplatin provided no additional benefit to the first-line treatment of advanced gastric cancer [21].

The REAL-3 study, a randomized phase 3 trial, compared panitumumab plus chemotherapy with chemotherapy in 553 patients with advanced esophageal and gastric cancer. However, the survival rate in the combination arm was lower than that in the chemotherapy-alone arm (PFS, 6.0 months vs. 7.4 months, P = 0.068; OS, 8.8 months vs. 11.3 months, P = 0.013) [22]. 

The overexpression of HER-2 occurs in 10%–30% of gastric cancers [23]. While HER-2 overexpression is associated with aggressive tumor biology in breast cancer [24], its prognostic importance in gastric cancer is less clear. It was suggested in early studies of gastric cancer that HER-2 had a negative effect on prognosis [9]. However, later studies did not find any prognostic effect of HER-2 [1]. Trastuzumab (a monoclonal antibody targeted against HER-2) causes antibody-dependent cellular cytotoxicity and inhibits HER-2 receptor signalization [9]. Trastuzumab is a standard therapy for early-stage and metastatic HER-2-positive breast cancers [9]. In the ToGA study, the OS for HER-2-positive gastric cancers was significantly improved when trastuzumab was used in combination with chemotherapy rather than chemotherapy alone. The overall HER-2 positivity rate was 22.1%, and the median OS was significantly improved in the trastuzumab group compared to the chemotherapy control group (13.8 vs. 11.1 months, P = 0.0046) [10]. Trastuzumab plus chemotherapy was indicated as a novel standard in HER-2-positive gastric and gastroesophageal junction tumors in the ToGA study. Masorini et al. showed positivity of EGFR and HER-2 in 75 (9%) and 113 (13.6%) patients, respectively. The overall and relapse-free survival rates were significantly lower in EGFR-positive patients compared to EGFR-negative patients, regardless of HER-2 status. EGFR positivity was associated with poor outcomes in multivariate analysis [1].

PTEN gene mutation is rarely seen in gastric cancer. However, loss of heterozygosity (LOH) is rather common [13]. The LOH rate is significantly higher in advanced gastric cancer than early-stage gastric cancer (63% and 18%, respectively) and is also significantly higher in poorly differentiated tumors than well-differentiated gastric tumors (69% and 29%, respectively) [13]. Zhu et al. stated in their study that the loss of cytoplasmic PTEN expression in gastric carcinoma was more common in comparison to the adjacent nonneoplastic tissues. The loss of cytoplasmic PTEN was correlated with histological stage. Total loss of PTEN expression or nuclear loss of PTEN was correlated with tumor grade. PTEN expression was not significantly related to the 3-year survival and OS rates [25]. Li et al. showed that the loss of cytoplasmic PTEN expression was associated with distant metastasis and advanced clinical stage in gastric cancer patients [14].

Cigarette smoking and alcohol consumption are considered potential risk factors for gastric cancer. The mechanism of how tobacco smoking increases the risk of gastric cancer is not well understood. The risk of gastric cancer development is 1.45 times higher in smokers than in nonsmokers [26]. Han et al. found an association between cigarette smoking after surgery and poor survival and cancer recurrence. However, they did not indicate a relationship between alcohol consumption and survival [27]. In certain studies, alcohol consumption was found to be significantly associated with poor outcomes in gastric cancer patients [28]. Cigarette smoking was an independent risk factor for DFS, but alcohol consumption was not associated with survival in our study. 

PNI is a poor prognostic factor in certain cancers, such as head/neck and prostate cancers [29]. It is also correlated with an aggressive tumor genotype [17]. The prognostic significance of PNI in gastric cancer has been investigated in several studies. Duroker et al. stated that PNI did not add any additional information to classic prognostic factors [30]. Bilici et al. showed that PNI (–) patients had longer survival than PNI (+) patients, and PNI was found to be a prognostic factor for the curative treatment of patients with gastric cancer [31]. In the study of Deng et al., PNI positivity was shown to be an independent prognostic factor affecting OS and DFS of patients with gastric cancer who underwent curative resection [17]. In our study, there was a significant difference between PNI (+) and PNI (-) patients in terms of OS. The median OS was 26.07 and 34.9 months in PNI (+) and PNI (-) patients, respectively (P = 0.042, log-rank).

The statistical power of our study is weak because distant metastasis developed in only 12 patients. The power of HER-2 as a prognostic factor was also weak, as HER-2 positivity was only seen in nine of 69 patients. 

In conclusion, this study suggests that IGF-1R, EGFR, and cigarette smoking can be used as additional prognostic factors to the previously known prognostic factors in gastric cancer patients treated with chemoradiation therapy. IGF-1R and EGFR may be used for patient selection in future prospective studies that evaluate the prognostic importance of these receptors. 

## Acknowledgments

This study was presented as a digital poster discussion session at the 56th ASTRO Annual Meeting, 14–17 September 2014, San Francisco, CA, USA. This project was supported by the Scientific and Research Council of Necmettin Erbakan University, Project Number 141618023.
